# Supplementary material for: Feeding Frequency Affects the Growth Performance and Intestinal Health of Juvenile Red-Tail Catfish (Hemibagrus wyckioides) with the Same Amount of Daily Feed
Source: Animals (Basel). 2025 May 30;15(11):1621. doi: 10.3390/ani15111621 (PMC12153739; doi:10.3390/ani15111621)

Supplementary materials

Table S1. Primers used in the study.

Gene	Accession No.	Forward (5' → 3')	Reverse (5' → 3')
Keap1	XM_058395007.1	GATAGCCTGAAGGTTCTGTGCG	AGGTGTTGTTTCGTGGGTCAAA
Nrf2	XM_058392117.1	GCCTATGCTTACCCAGAATCCC	GGCAGATACTGGCTGTAGTTGGA
Sod	XM_058382267.1	CGTGACCGCCAATTCCGATG	CACCACAAGCCAGACGACCT
Cat	XM_058408555.1	TCCGTCCTTCATCCACTCTCA	GTCCATCAGGCAATCCACGAT
Gpx	XM_058382267.1	GCATCACCTCGCTGTATCTCC	TCGCCACATTCACAACCAGAG
β-actin	XM_058397995.1	AGAGGTATCCTGACCCTGAAGTAC	GAGCATAACCTTCATAGATGGGCACAG

Figure S1. Diagram of the three-dimensional circulating water culture tanks using in this study. A, the fish were fed twice per day at 8:00 and 20:00; B, the fish were fed three times per day at 8:00, 14:00, and 20:00; C, the fish were fed four times per day at 9:00, 12:00, 16:00, and 20:00. EWS, external water system; WSS, water-supply system; WPS, water purification system. The black arrows indicate the direction of water flow.

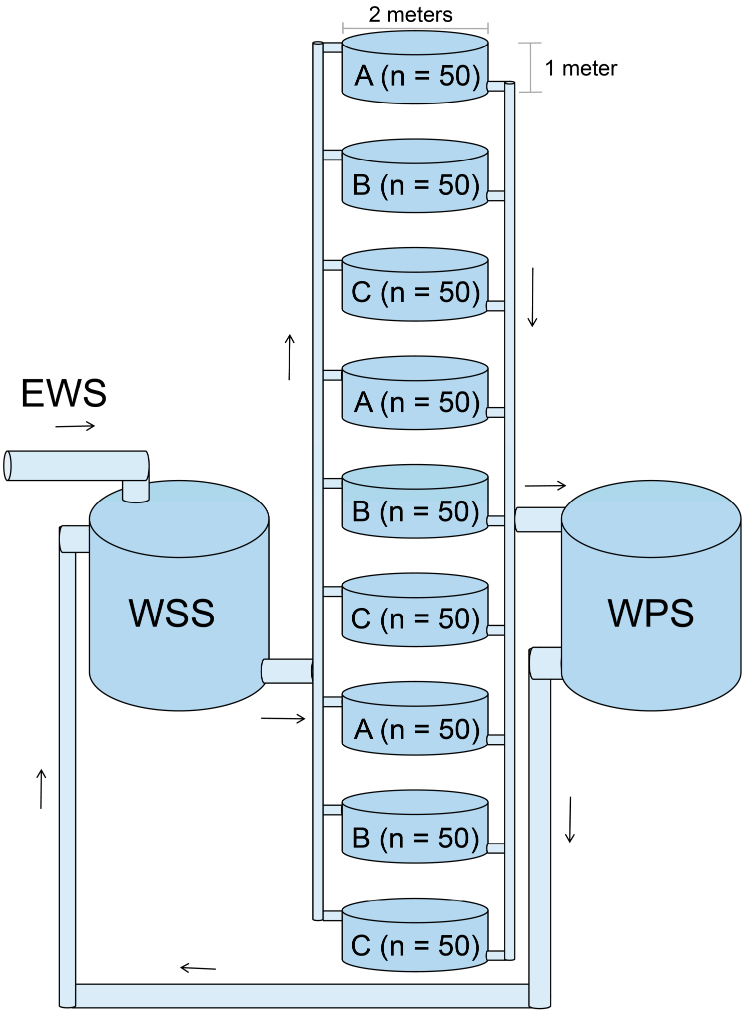

Figure S2. Potential regulatory patterns in *H. wyckioides*. (A) A correlation analysis based on the Spearman correlation coefficient was performed to examine the interactions between the module eigengenes extracted from the matrix of 13 differentially abundant ASVs and the secondary module eigenvectors extracted from the phenotype matrix. (B) The positions of differentially abundant microorganisms and selected key microorganisms in the evolutionary tree are shown. (C) Boxplot of the identified biomarkers.

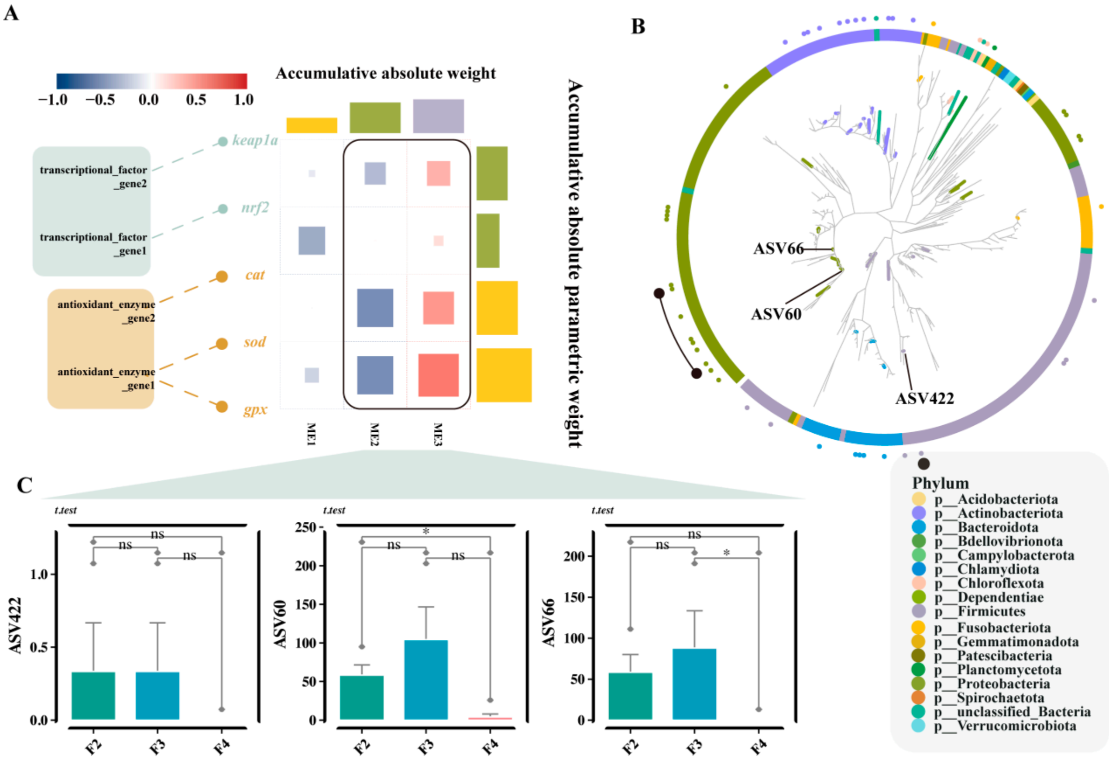

Figure S3. Molecular ecological network analysis of the intestinal microbiota of *H. wyckioides*. (A) Community assembly process evaluation based on the neutral community model. A higher R2 represents a better fit of the neutral model, which means a greater contribution of stochastic processes to the construction of the community. (B) Circos plot for visualizing microbial interactions. The first row is plotted based on microbial species and the second row is plotted based on abundance. The bands are, from outside to inside, phylum, class, order, family, genus, and species. Red edges represent positively correlated microbial interactions. Green edges represent positively correlated microbial interactions.

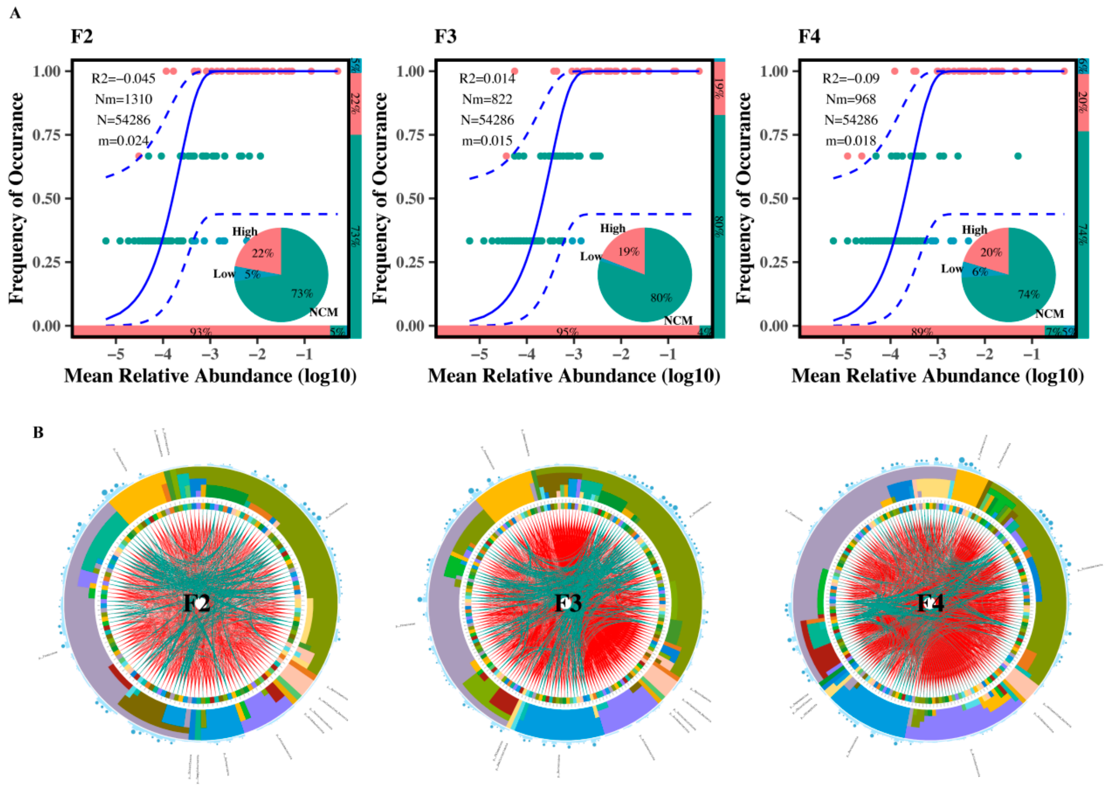

Figure S4. Modularity and ecological roles from the molecular ecological network analysis. Specific modules of microbial networks were extracted, and the abundance of the microbial composition at the phyla level is highlighted. Moreover, modules represent assemblages of microbes that can perform specific ecological functions. The modules of the microbial network from each group were analyzed for the similarity of modules by Fisher's test, and modules were considered similar if the differences were significant. Similar categories of modules were grouped into module clusters and colored accordingly. This analysis allowed the retention of microbial network modules and the potential ecological functions that could be important under the different feeding frequencies.

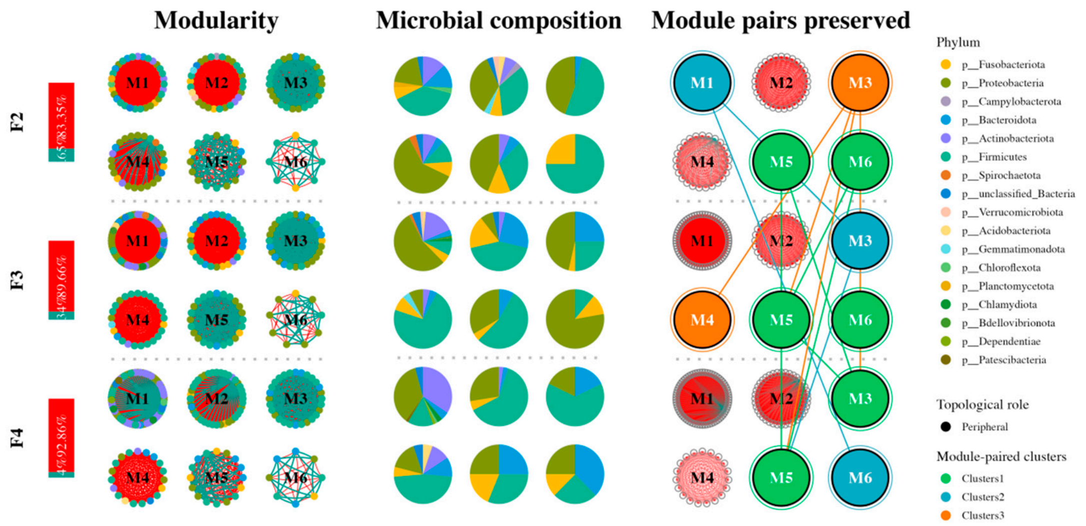

Supplement: Supplementary file 1 [file animals-15-01621-s001.zip › animals-3644043-supplementary.pdf]
